# Supplementary material for: Discovery of novel microRNA mimic repressors of ribosome biogenesis
Source: Nucleic Acids Res. 2024 Jan 9;52(4):1988–2011. doi: 10.1093/nar/gkad1235 (PMC10899765; doi:10.1093/nar/gkad1235)
Supplement: gkad1235_Supplemental_Files [file gkad1235_supplemental_files.zip › microRNA_ms_supplementary_data_final.pdf]

Discovery of novel microRNA mimic repressors of ribosome biogenesis

Supplementary Materials

Carson J. Bryant [a], Mason A. McCool [a], Gabriela T. Rosado-González [a], Laura Abriola [b], Yulia V. Surovtseva [b], Susan J. Baserga [a, c, d]

[a] Department of Molecular Biophysics and Biochemistry, Yale School of Medicine, New Haven, CT, USA.

[b] Yale Center for Molecular Discovery, Yale University, West Haven, CT, USA.

[c] Department of Genetics, Yale School of Medicine, New Haven, CT, USA.

[d] Department of Therapeutic Radiology, Yale School of Medicine, New Haven, CT, USA.

Address correspondence to Susan J. Baserga (Molecular Biophysics and Biochemistry, PO Box 208024, 333 Cedar Street, New Haven, CT 06520-8024, USA; +1-203-785-4618, susan.baserga@yale.edu).

Supplementary Figure 1

A

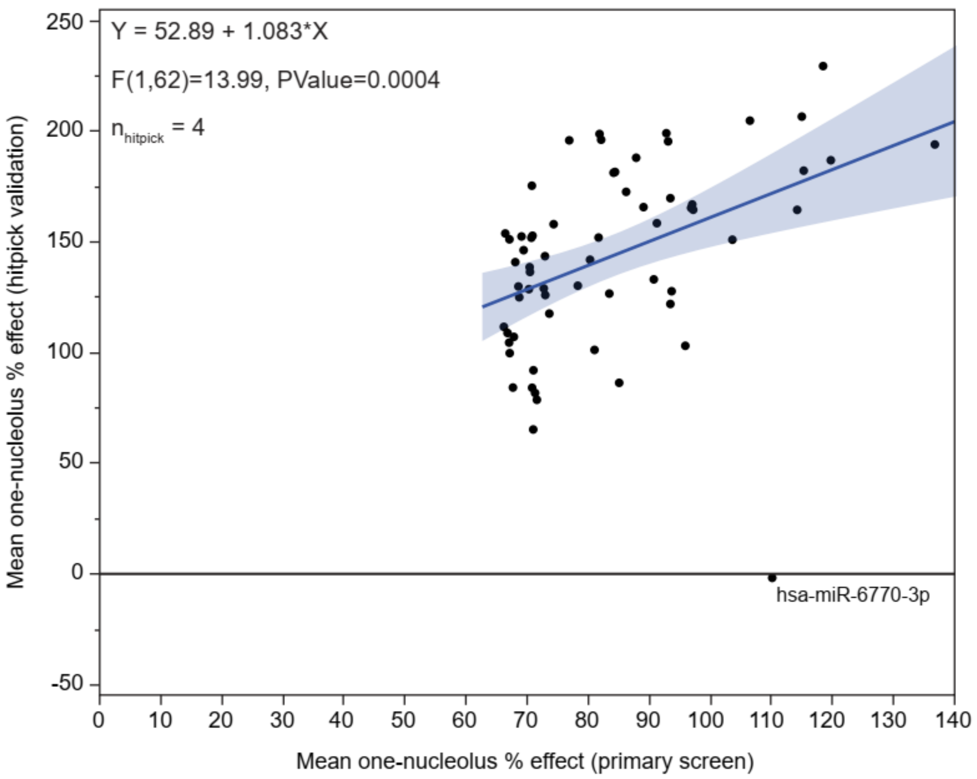

B

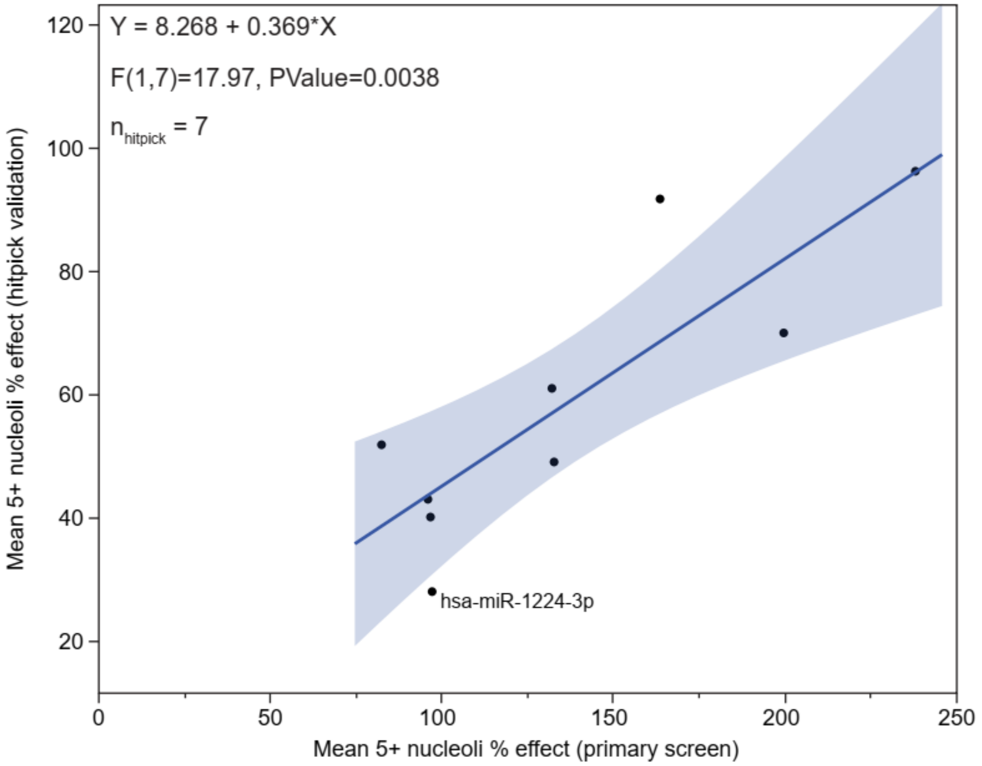

Supplementary Figure 1. Primary screen hitpick validation excludes hsa-miR-6770-3p and hsa-miR-1224-3p.

(**A**) Comparison of mean one-nucleolus percent effects from primary screen (x-axis) or hitpick validation (y-axis) for 64 one-nucleolus hits. The one-nucleolus hitpick was conducted with $n = 4$ replicates. Graphing and linear regression was performed in JMP. hsa-miR-6770-3p was excluded from downstream analysis.

(**B**) Comparison of mean 5+ nucleoli percent effects from primary screen (x-axis) or hitpick validation (y-axis) for nine 5+ nucleoli hits. The 5+ nucleoli hitpick was conducted with $n = 7$ replicates. Graphing and linear regression was performed in JMP. hsa-miR-1224-3p was excluded from downstream analysis.

Supplementary Figure 2

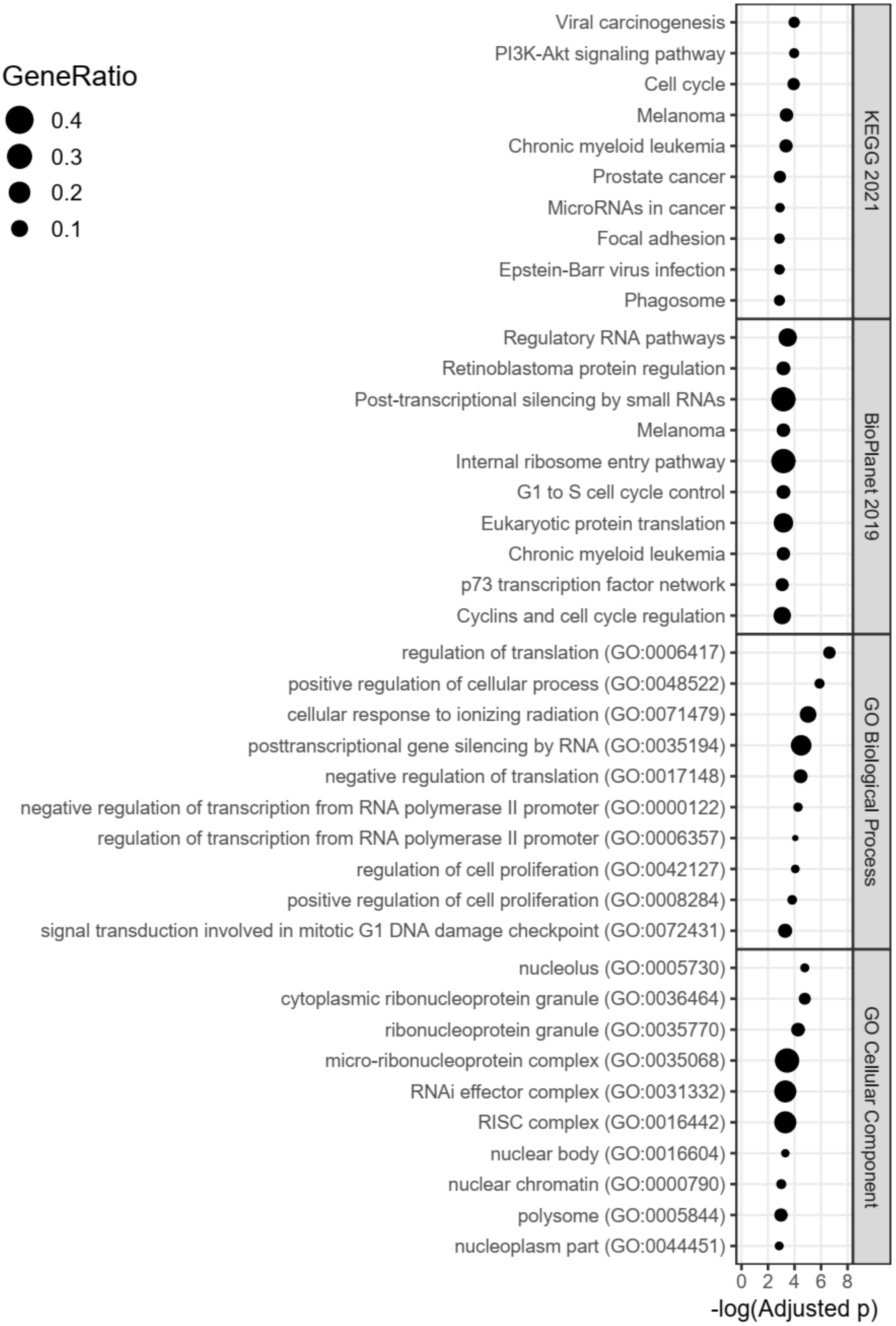

Supplementary Figure 2. Targets of 24 MirGeneDB hits represented in TarBase 8 are enriched for regulators of RNA pathways, translation, the cell cycle, and for localization in the nucleolus.

Enrichment plots for 135 genes targeted by 5 or more of the microRNA hits present in MirGeneDB. Plots indicate $-\log_{10}$(adjusted *p*) on the x-axis and the gene ratio as the marker size. Enrichment analysis was conducted with Enrichr, and plots were made in R. Enrichment databases: Kyoto Encyclopedia of Genes and Genomes (KEGG) 2021; NCATS BioPlanet of Pathways 2019; Gene Ontology (GO) Biological Process 2018; GO Cellular Component 2018.

Supplementary Figure 3

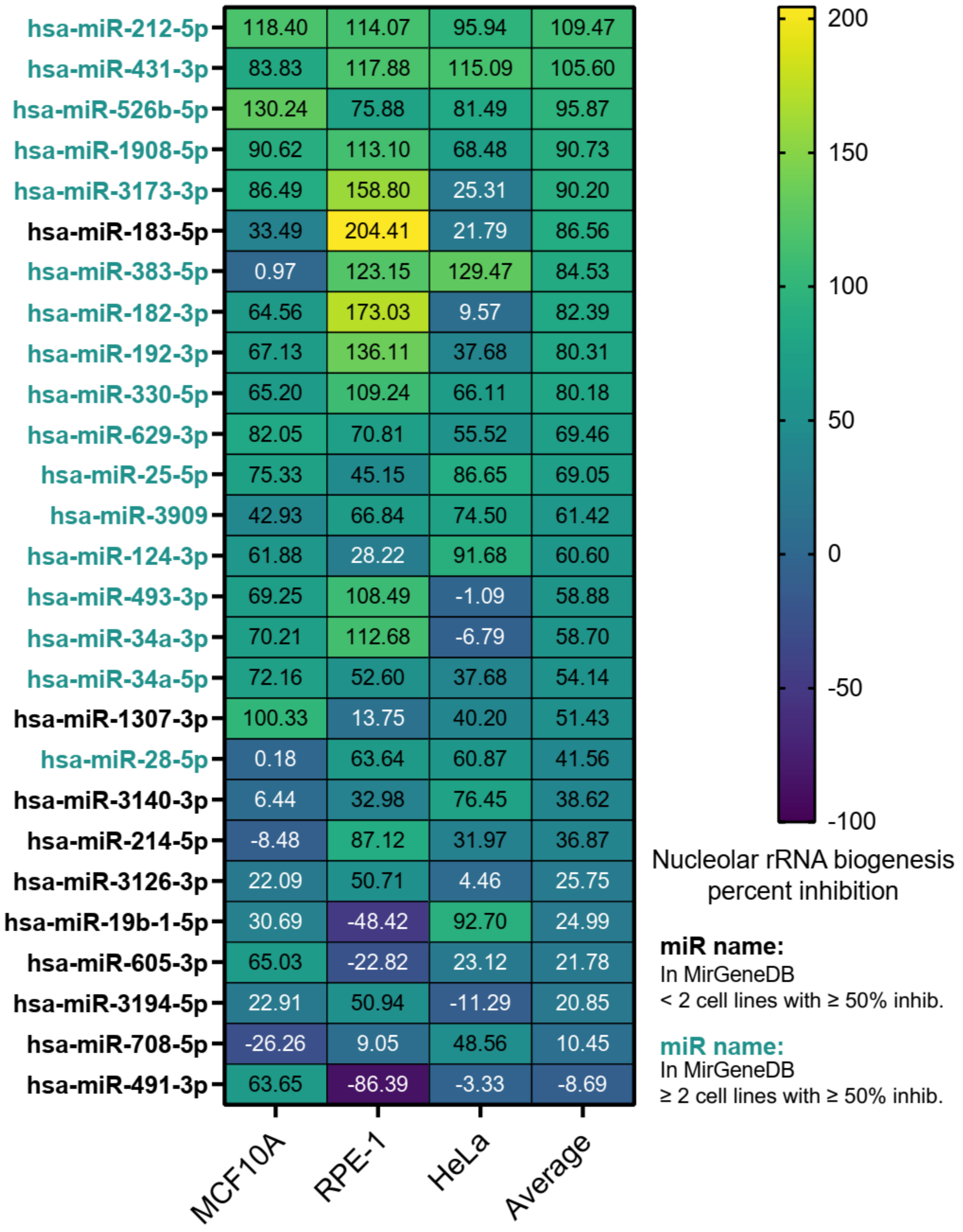

Supplementary Figure 3. Conservation of nucleolar rRNA biogenesis inhibition by MirGeneDB microRNA hits across 3 human cell lines.

Heatmap showing the nucleolar rRNA biogenesis percent inhibition following overexpression of 27 microRNA mimic hits present in MirGeneDB in 3 diverse human cell lines. Cell types are indicated on the x-axis, with an unweighted average column also calculated. All microRNA hit names are bolded because each microRNA is present in MirGeneDB. MicroRNA hit names are colored depending on conservation across cell lines. Hits whose overexpression causes at least a 50% inhibition of nucleolar rRNA biogenesis in two or more cell lines (teal name); hits failing this criterion (black name).

Supplementary Figure 4

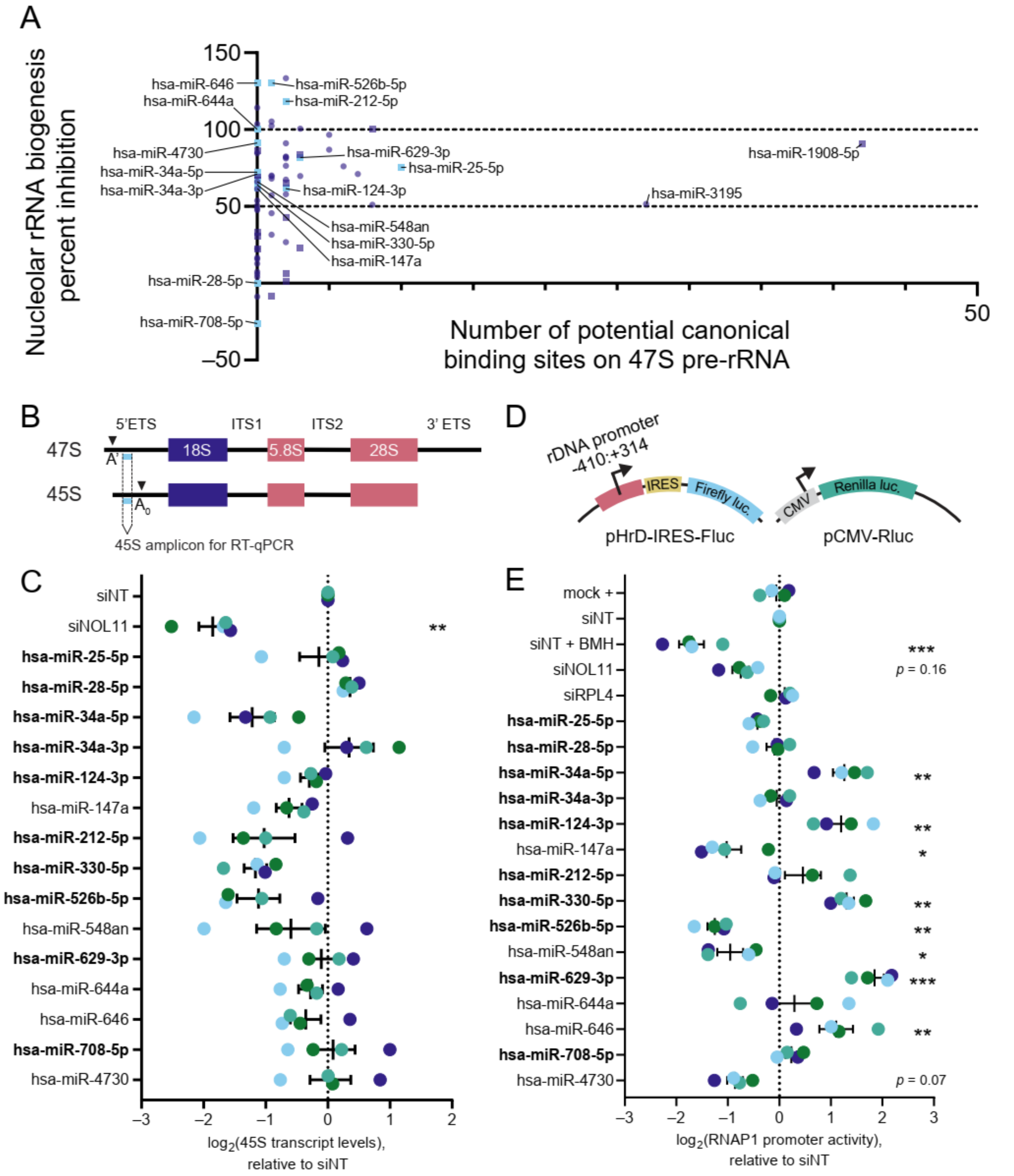

Supplementary Figure 4. MicroRNA hits do not reliably alter RNAP1 transcription.

(**A**). Scatter plot comparing the number of potential canonical (seed) binding sites on the 47S pre-rRNA transcript to the nucleolar rRNA biogenesis percent inhibition for the 72 microRNA hits, as predicted by BLAST. A number of hits have close to 0 predicted canonical 47S binding sites but strong nucleolar rRNA biogenesis percent inhibition. Select hits are labeled, and labels for percent inhibition are shown at 50% inhibition (empirical assay cutoff, consistent with pre-rRNA modification defect) and 100% inhibition (siPOLR1A positive control, consistent with pre-rRNA transcription defect) (55). The data were graphed in GraphPad Prism 8.

(**B**). Schematic indicating the amplicon (light blue) used for 45S pre-rRNA RT-qPCR, located between the A' and A_0 cleavage sites in the 5' ETS of the primary rRNA transcript. The location of the mature 18S, 5S and 28S rRNAs are indicated.

(**C**). RT-qPCR analysis of levels of the 45S pre-rRNA precursor transcript as a proxy for RNAP1 transcription. Hit names are bolded for MirGeneDB members. The mean ± SEM are shown alongside individual data points, colored by replicate (4 replicates). The data were normalized to 7SL RNA abundance as an internal control, then to siNT for comparison using the $\Delta\Delta C_T$ method. The data were analyzed by ordinary one-way ANOVA with multiple comparisons against siNT and Holm-Šídák correction in GraphPad Prism 8. *****, $p < 0.05$; ******, $p < 0.01$; *******, $p < 0.001$.

(**D**). Schematic for the dual-luciferase reporter assay for RNAP1 promoter activity (54,99). MCF10A cells were co-transfected with pHrD-IRES-Fluc, in which a 724 bp fragment of the rDNA promoter and early 5' ETS drive firefly luciferase production, and pCMV-Rluc, in which the constitutive CMV promoter drives *Renilla* luciferase production.

(**E**). Dual-luciferase reporter assay for RNAP1 promoter activity. Hit names are bolded for MirGeneDB members. The mean ± SEM are shown alongside individual data points, colored by replicate (4 replicates). The data were analyzed by ordinary one-way ANOVA with multiple comparisons against siNT and Holm-Šídák correction in GraphPad Prism 8. *****, $p < 0.05$; ******, $p < 0.01$; *******, $p < 0.001$.

Supplementary Figure 5

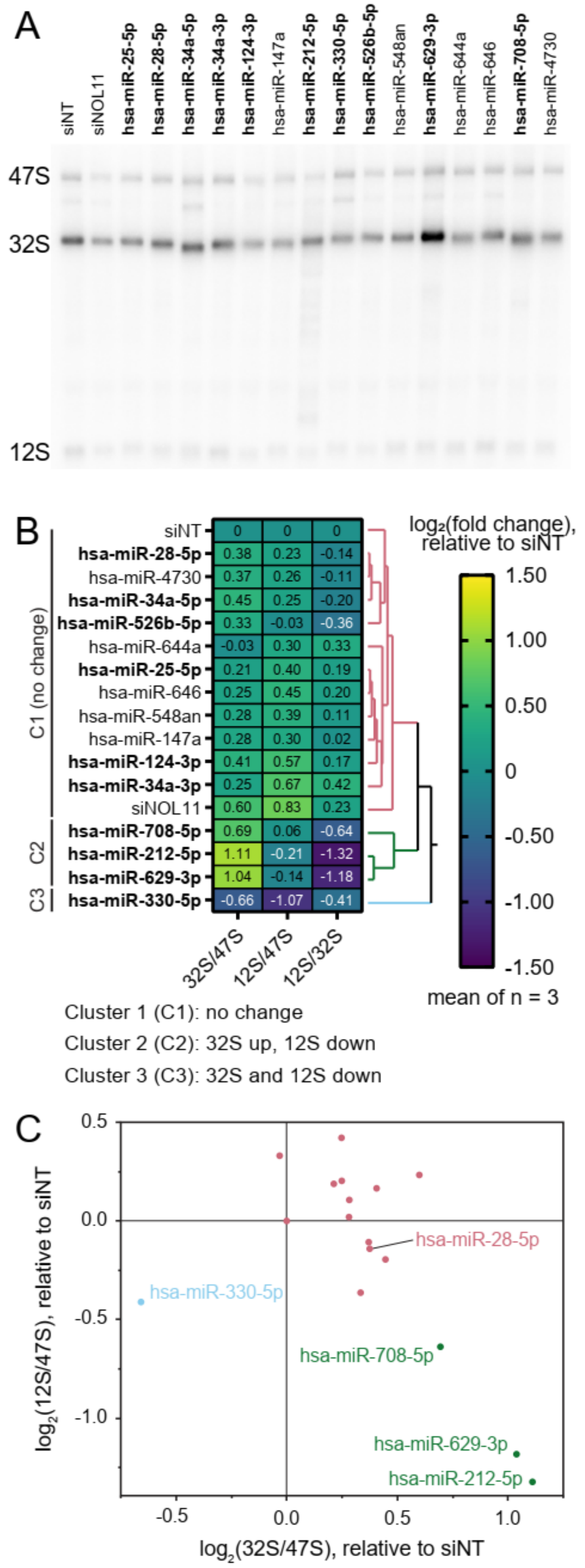

Supplementary Figure 5. Four microRNA hits slightly interfere with ITS2 processing.

(**A**). Representative ITS2 (probe P4) northern blot of 3 µg of total RNA isolated from control- or hit-treated MCF10A cells. The pre-rRNA processing intermediates are labeled on the left according to the schematic in Figure 5A. The images were quantified using Bio-Rad Image Lab. Hit names are bolded for MirGeneDB microRNAs.

(**B**). Clustered heatmap showing $\log_2$-transformed Ratio Analysis of Multiple Precursor [RAMP, (77)] calculations for microRNA mimic hits, normalized to si non-targeting (siNT) negative control. Values represent mean $\log_2$-scale RAMP ratio for $n = 3$ replicates. Clusters: no change (C1, red); 32S up, 12S down (C2, green); both 32S and 12S down (C3, blue). The RAMP ratios were calculated in Microsoft Excel. Three clusters were assigned using hierarchical Ward clustering in JMP, and data were graphed in GraphPad Prism 8. Hit names are bolded for MirGeneDB microRNAs.

(**C**). Log_2-scale 32S/47S and 12S/47S pre-rRNA precursor RAMP ratios after subset microRNA mimic treatment, relative to a non-targeting (siNT). Cluster colors the same as in (**B**). The data were graphed in GraphPad Prism 8, with select hits labeled.

Supplementary Figure 6

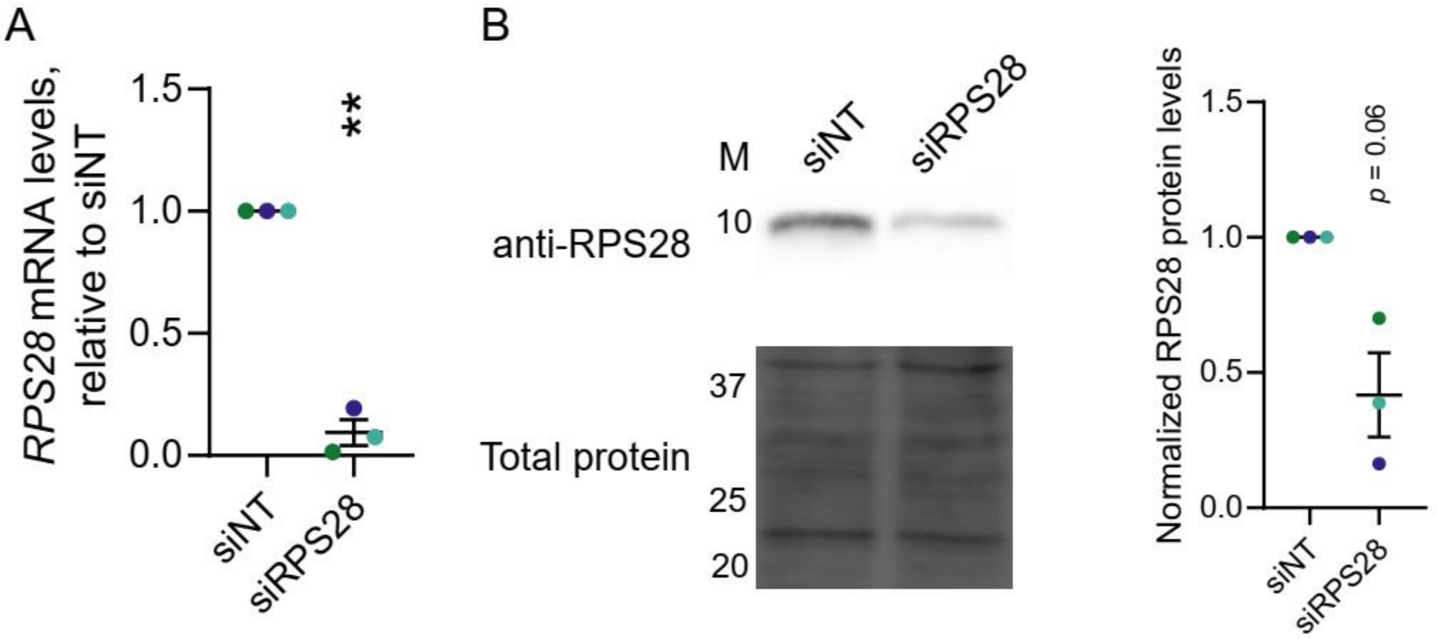

Supplementary Figure 6. Validation of siRNA-mediated knockdown of RPS28.

(**A**) RT-qPCR quantification of mRNA transcript levels following siRNA knockdown of *RPS28* in MCF10A cells. The data were normalized to 7SL RNA abundance as an internal control, then to a non-targeting siRNA (siNT) for comparison using the $\Delta\Delta C_T$ method. The mean ± SEM are shown alongside individual data points, colored by replicate (3 replicates). Data were analyzed by unpaired two-sided Welch's *t*-tests in GraphPad Prism 8. *****, $p < 0.05$; ******, $p < 0.01$; *******, $p < 0.001$.

(**B**) Immunoblot quantification of protein levels following siRNA knockdown for *RPS28* in MCF10A cells. Anti-RPS28 blotting is shown in the example immunoblot. Total protein is the trichloroethanol total protein stain loading control. M, molecular weight marker lane in kDa. The images were quantified using Bio-Rad Image Lab. The mean ± SEM are shown alongside individual data points, colored by replicate (3 replicates). The data were graphed and analyzed by unpaired two-sided Welch's *t*-tests in GraphPad Prism 8. *****, $p < 0.05$; ******, $p < 0.01$; *******, $p < 0.001$.

Supplementary Figure 7

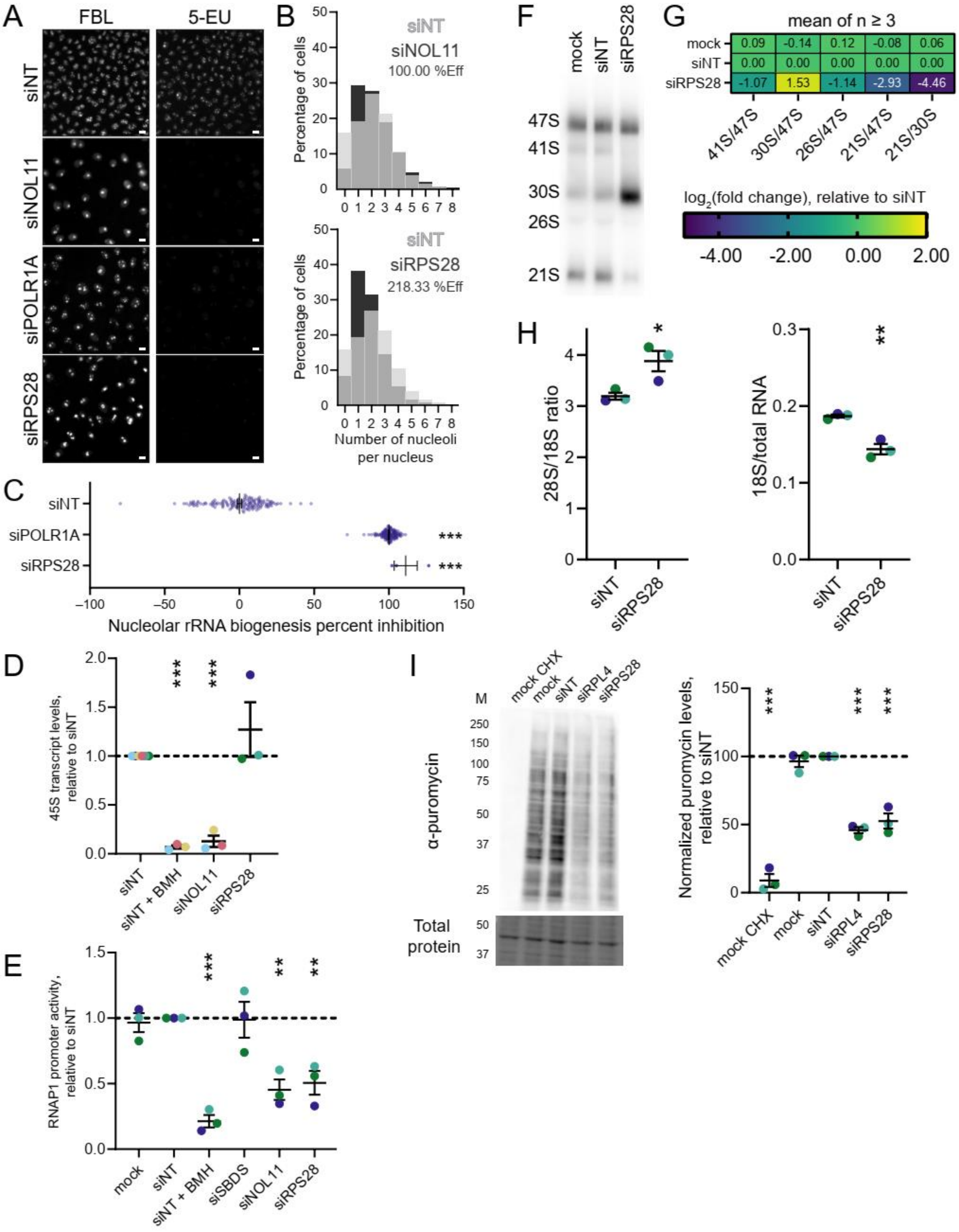

Supplementary Figure 7. RPS28 knockdown decreases nucleolar number, inhibits nucleolar rRNA biogenesis, interdicts pre-18S processing, and reduces global protein synthesis.

(**A**). Representative images of control-treated or RPS28-depleted MCF10A cells following fibrillarin (FBL) antibody staining and 5-EU incorporation. FBL immunostaining and 5-EU click labeling are shown as separate channels. Scale bars, 10 µm. siNT is a non-targeting negative control siRNA. siNOL11, NOL11 knockdown is a positive control for the one-nucleolus phenotype. siPOLR1A, POLR1A (RPA194) knockdown is a positive control for nucleolar rRNA biogenesis inhibition. siRPS28, RPS28 knockdown.

(**B**) Quantification of nucleolar number following siRNA depletion of RPS28. The histograms indicate the percentage of cells with a given number of nucleoli. The siNT negative control histogram is shown in light gray on all graphs for reference. Histograms from cells depleted of NOL11 or RPS28 are shown in black. siNOL11 is the positive control for decrease in nucleolar number. The one-nucleolus percent effect for black-labeled treatment is shown.

(**C**). Quantification of the percent inhibition of nucleolar rRNA biogenesis. The overall mean percent inhibition ± SEM is shown for each treatment, with each dot representing one well. The siNT negative control is set to 0% inhibition, and the siPOLR1A positive control is set to 100% inhibition. Data were analyzed by ordinary one-way ANOVA with multiple comparisons against siNT and Holm-Šídák correction in GraphPad Prism 8. ******* $p < 0.001$.

(**D**). RT-qPCR analysis of levels of the 45S pre-rRNA as a proxy for RNAP1 transcription. siNT, non-targeting siRNA negative control. siNT + BMH, siNT transfection plus acute 1 µM BMH-21 pre-treatment to selectively inhibit RNAP1 activity. siNOL11, NOL11 depletion as a positive control. siRPS28, RPS28 depletion. The mean ± SEM are shown alongside individual data points, colored by replicate (3 replicates). The data were normalized to 7SL RNA abundance as an internal control, then to siNT for comparison using the $\Delta\Delta C_T$ method. Data were analyzed by ordinary one-way ANOVA with multiple comparisons against siNT and Holm-Šídák correction in GraphPad Prism 8. *******, $p < 0.001$.

(**E**). Dual-luciferase reporter assay for RNAP1 promoter activity (54,99). Mock, no siRNA mock transfection control. siNT, non-targeting siRNA negative control. siNT + BMH, siNT transfection plus acute 1 µM BMH-21 pre-treatment to selectively inhibit RNAP1 activity. siSBDS, depletion of the cytoplasmic pre-60S assembly factor SBDS as a negative control. siNOL11, NOL11 depletion as a positive control. siRPS28, RPS28 depletion. The mean ± SEM are shown alongside individual data points, colored by replicate (3 replicates). The data were analyzed by ordinary one-way ANOVA with multiple comparisons against siNT and Holm-Šídák correction in GraphPad Prism 8. ******, $p < 0.01$; *******, $p < 0.001$.

(**F**). Representative ITS1 (probe P3) northern blot of 3 µg of total RNA isolated from RPS28-depleted MCF10A cells. Mock, no siRNA mock transfection control. siNT, non-targeting siRNA negative control. siRPS28, RPS28 depletion. The pre-rRNA processing intermediates are labeled on the left. The images were quantified using Bio-Rad Image Lab.

(**G**). Heatmap showing $\log_2$-transformed Ratio Analysis of Multiple Precursor [RAMP (77)] calculations, normalized to the si non-targeting (siNT) negative control. The values represent mean RAMP ratio for $n = 4$ replicates, except $n = 3$ for mock. RAMP ratios were calculated in Microsoft Excel and data were graphed in GraphPad Prism 8.

(**H**). Bioanalyzer analysis for 1 µg of total RNA isolated from RPS28-depleted MCF10A cells. Left, the 28S/18S mature rRNA ratio; right, the 18S mature rRNA/total RNA ratio. Mean ± SEM are shown alongside individual data points, colored by replicate (3 replicates). The data were graphed and analyzed by ordinary one-way ANOVA with multiple comparisons against a non-targeting siRNA (siNT) and Holm-Šídák correction in GraphPad Prism 8. *****, $p < 0.05$; ******, $p < 0.01$.

(**I**). Representative examples of SUnSET puromycin incorporation assay (53,80) immunoblots of total protein isolated from RPS28-depleted MCF10A cells. α-puromycin is an example blot for puromycin incorporation as a proxy for global protein synthesis. Total protein is the trichloroethanol total protein stain loading control. M, molecular weight marker in kDa. Mock, no siRNA mock transfection control. Mock CHX is mock cells co-treated for 1 h with puromycin and 100 µg/mL cycloheximide to halt protein translation. siNT, non-targeting siRNA negative control. siRPL4, depletion of the 60S component RPL4 as a positive control. siRPS28, RPS28 depletion. Images were quantified with Bio-Rad Image Lab. The mean ± SEM are shown alongside individual data points, colored by replicate (3 replicates). Data were normalized to a non-targeting siRNA (siNT), then graphed and analyzed by ordinary one-way ANOVA with multiple comparisons against siNT and Holm-Šídák correction in GraphPad Prism 8. *******, $p < 0.001$.

Supplementary Figure 8

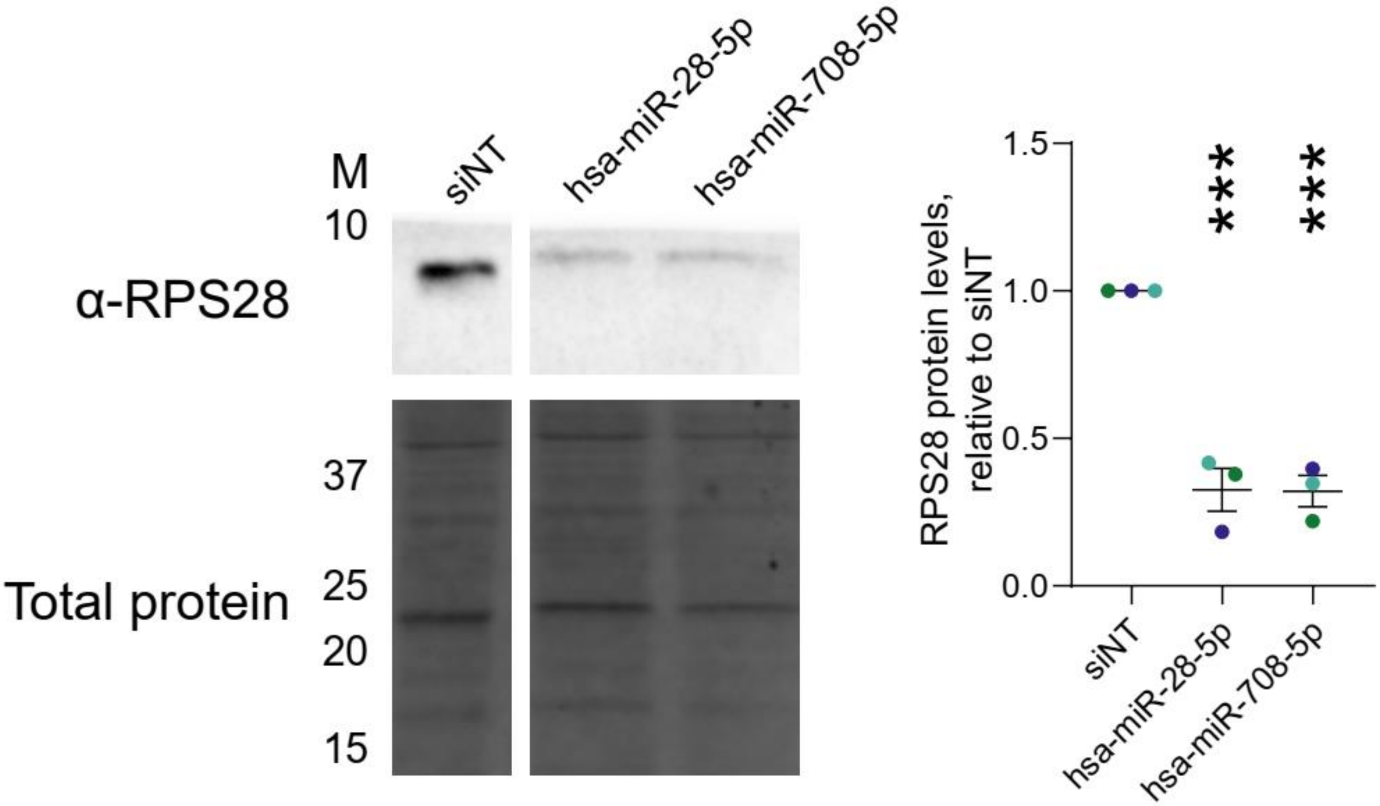

Supplementary Figure 8. RPS28 protein levels are potently by the MIR-28 siblings, hsa-miR-28-5p and hsa-miR-708-5p, in MCF10A cells.

Immunoblot analysis and quantification of RPS28 protein levels from control- or MIR-28 sibling-treated MCF10A cells. α-RPS28 indicates the example immunoblot for the labeled protein. Total protein, trichloroethanol total protein stain loading control. M, molecular weight marker lane in kDa. siNT, non-targeting siRNA negative control. hsa-miR-28-5p or hsa-miR-708-5p, transfected MIR-28 microRNA mimics. The images were quantified with Bio-Rad Image Lab. Mean ± SEM are shown alongside individual data points, colored by replicate (3 replicates). The data were normalized to siNT, then graphed and analyzed by ordinary one-way ANOVA with multiple comparisons against siNT and Holm-Šídák correction in GraphPad Prism 8. *******, $p < 0.001$.

Supplementary Figure 9

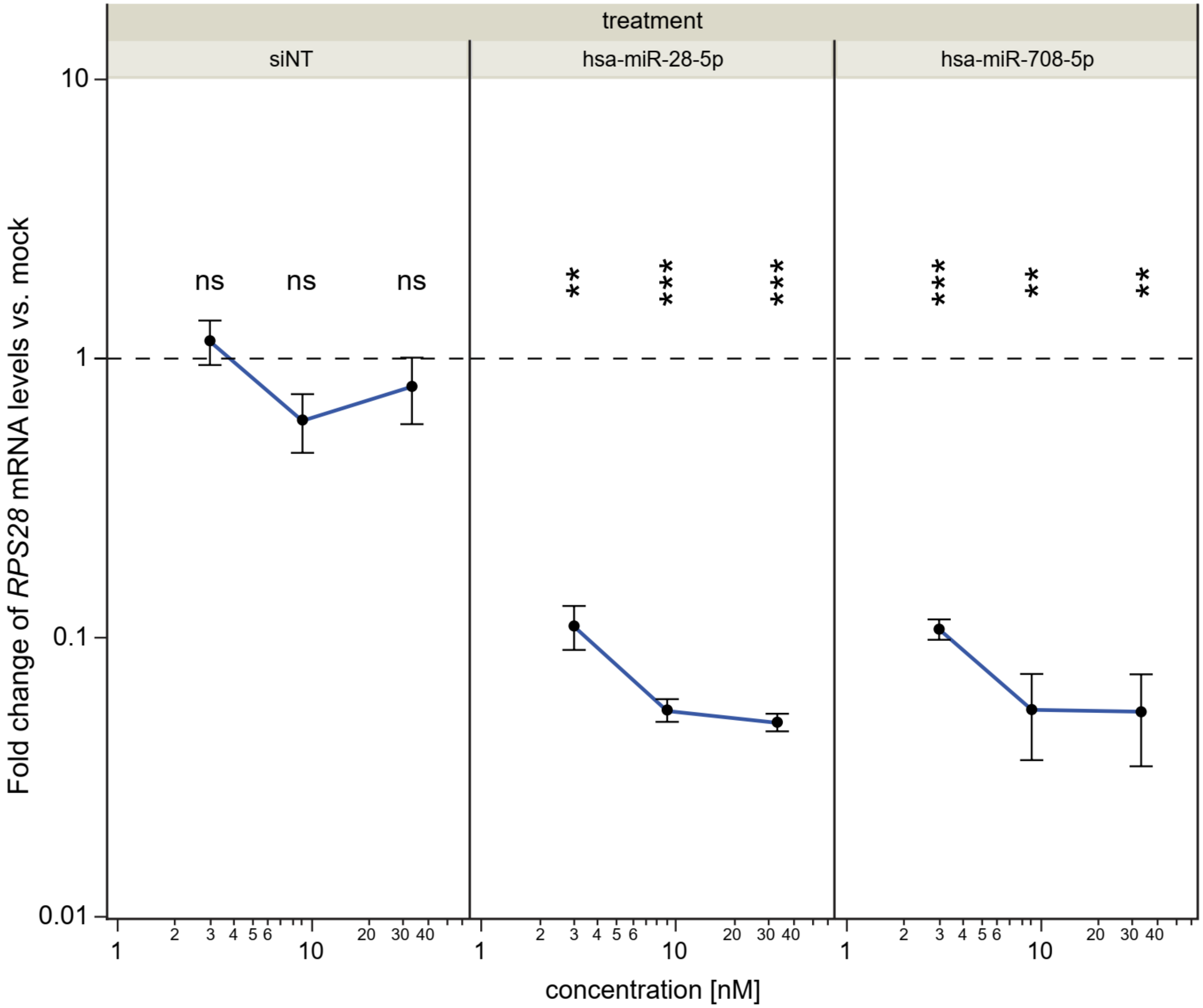

Supplementary Figure 9. ***RPS28*** **mRNA levels are reduced by the MIR-28 siblings, hsa-miR-28-5p and hsa-miR-708-5p, in hTERT RPE-1 cells.**

Three-point concentration response curves quantifying the MIR-28 siblings' effect on *RPS28* mRNA levels in hTERT RPE-1 cells by RT-qPCR. siNT, non-targeting siRNA negative control. Hsa-miR-28-5p or hsa-miR-708-5p, transfected MIR-28 microRNA mimics. Concentrations of siRNA/microRNA mimics were 3 nM, 9 nM and 33 nM. Fold change of *RPS28* mRNA levels compared to mock transfection is shown on the y-axis. Dashed line indicates the relative *RPS28* mRNA levels in the mock sample. The data were normalized to 7SL RNA abundance as an internal control, then to mock for comparison using the $\Delta\Delta C_T$ method. Data are graphed as mean ± SEM for $n = 3$ biological replicates and $n = 2$ technical replicates. Data were analyzed by ordinary one-way ANOVA with multiple comparisons against mock and Holm-Šídák correction in GraphPad Prism 8. ns, no significance; **, $p < 0.01$; *******, $p < 0.001$.

Supplementary Figure 10

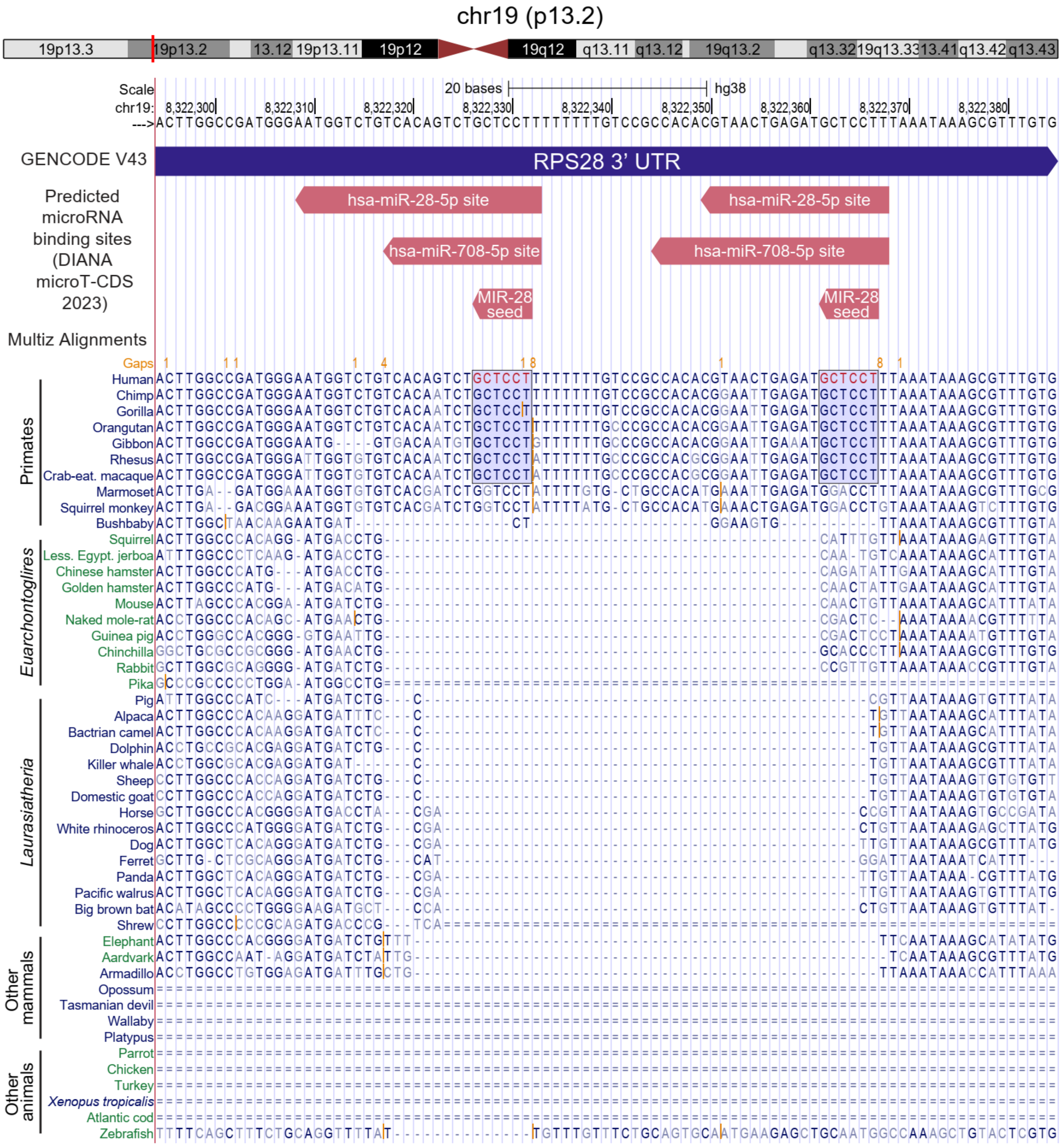

Supplementary Figure 10. The predicted tandem MIR-28 family binding sites in the RPS28 3' UTR are conserved only within a subset of primates.

UCSC Genome Browser visualization of the *RPS28* 3’ UTR region containing predicted tandem MIR-28 family binding sites. A schematic of the locus on human chromosome 19 is shown above the Browser view. A DIANA microT-CDS 2023 track with predicted binding sites and the MIR-28 family seed sequence is shown in red. Multi-species genomic alignments for selected species in the Multiz 100 Vertebrate Species Alignment and Conservation track are shown, with group labels annotated on the left. Conservation of the predicted 6mer MIR-28 sites within a subset of primates is highlighted with a light blue box.

Supplementary Figure 11

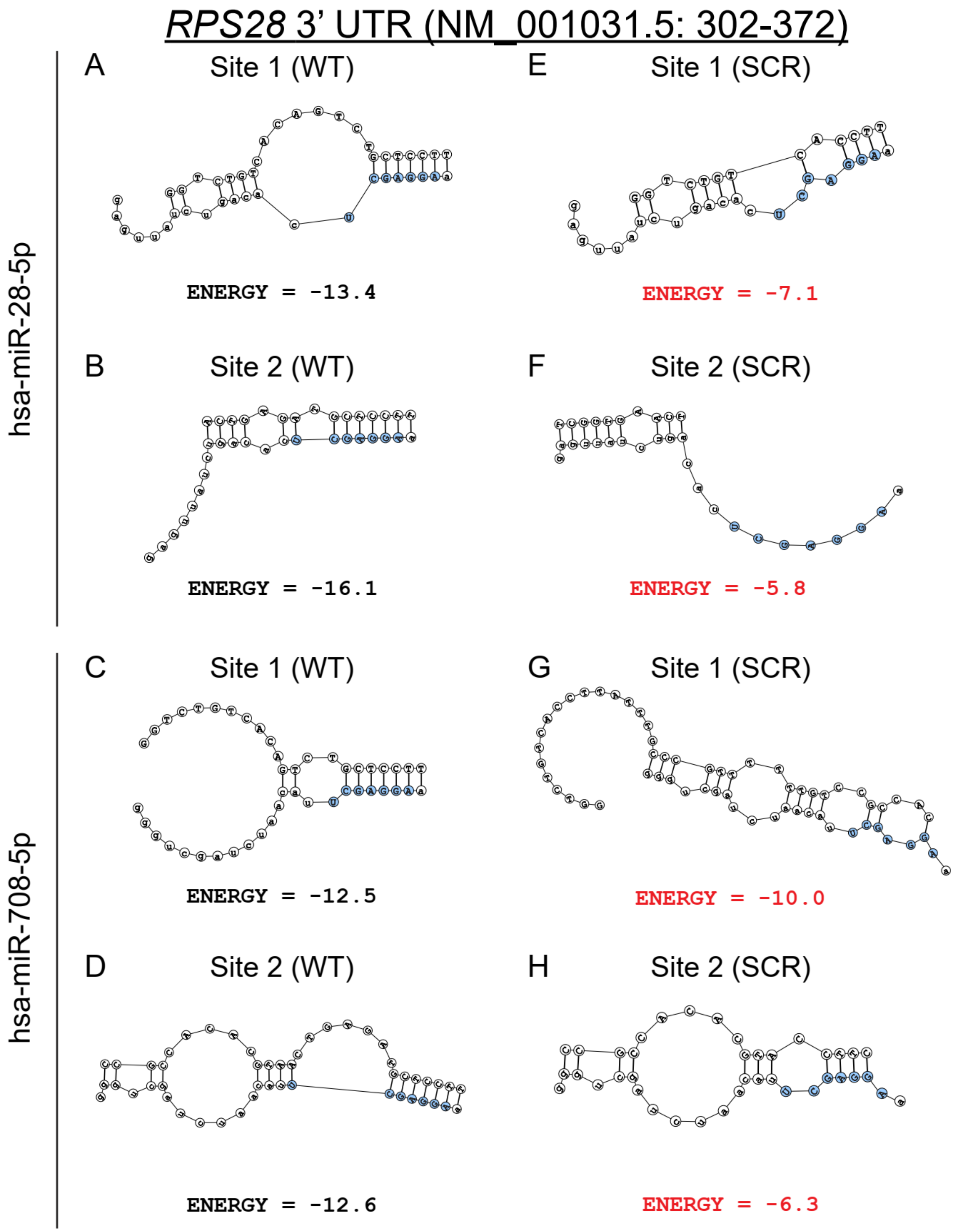

Supplementary Figure 11. Predicted binding structures between MIR-28 siblings and putative binding sites in the *RPS28* 3' UTR.

(**A-D**). DuplexFold predicted structures between hsa-miR-28-5p (**A-B**) or hsa-miR-708-5p (**C-D**) to wild-type (WT) putative MIR-28 sites in the *RPS28* 3' untranslated region (UTR). The seed sequence for each microRNA is shown in blue. The DuplexFold predicted binding energies are indicated for each structure. Transcriptomic coordinates for the binding region input are shown in the header. Extraneous target base pairs were removed for visualization.

(**E-H**). Corresponding DuplexFold predicted structures between hsa-miR-28-5p (**E-F**) or hsa-miR-708-5p (**G-H**) to seed site scrambled (SCR) putative MIR-28 sites in *RPS28* 3' untranslated region (UTR). Each binding energy (red) is reduced following seed scrambling. Extraneous target base pairs were removed for visualization.

Supplementary Figure 12

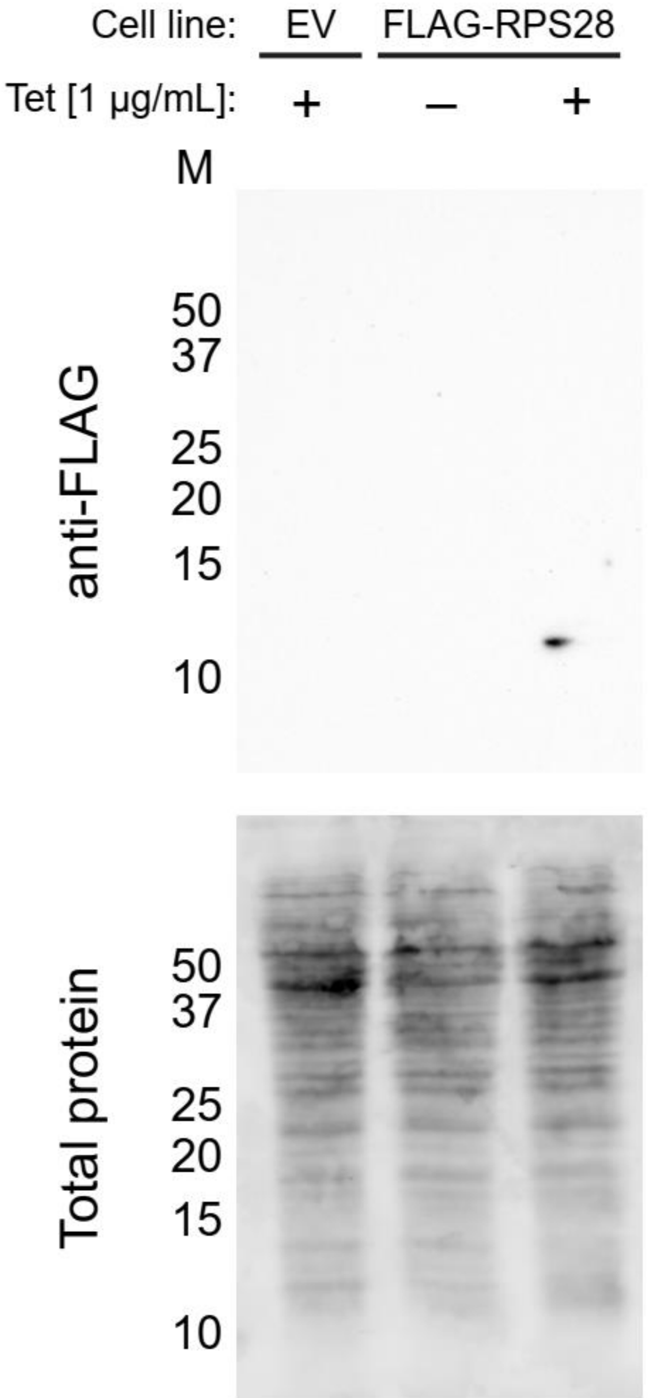

Supplementary Figure 12. Tetracycline-inducible expression of FLAG-tagged RPS28 in an engineered HEK 293 Flp-In T-REx cell line.

Immunoblot analysis of FLAG-tagged RPS28 protein levels from HEK 293 Flp-In T-REx cells. Anti-FLAG indicates an immunoblot for the FLAG tag. Total protein, trichloroethanol total protein stain loading control. M, molecular weight marker lane in kDa. Cell line, parental non-engineered/empty vector (EV) or FLAG-RPS28 engineered HEK 293 Flp-In cells. Tet, tetracycline induction of protein expression at 1 µg/mL for 48 h. +, positive tetracycline induction; –, no tetracycline induction.

Supplementary Figure 13

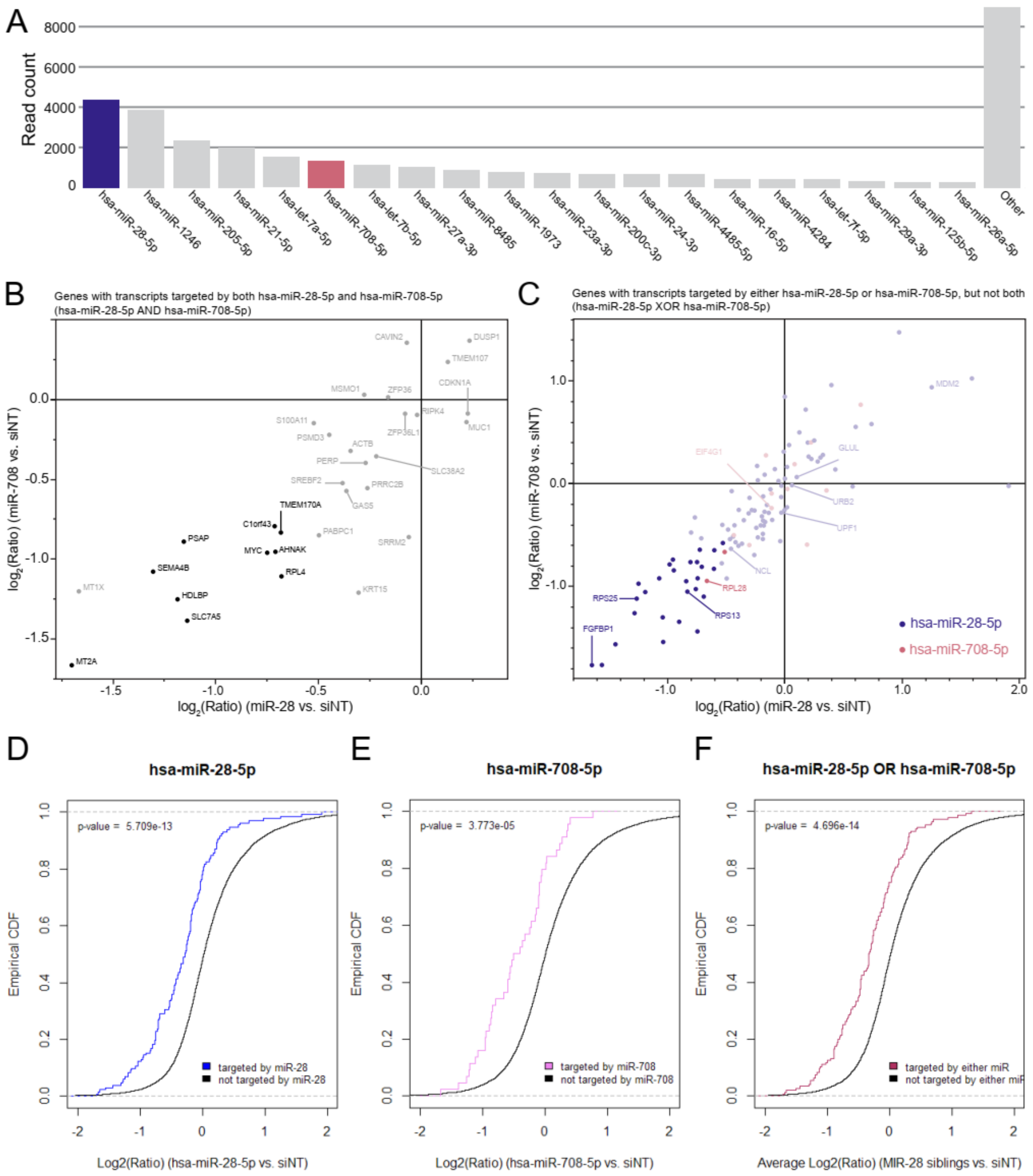

Supplementary Figure 13. Direct targets of the MIR-28 siblings revealed by miR-eCLIP.

(**A**). Read counts of microRNAs sequences enriched following MIR-28 mimic overexpression and miR-eCLIP AGO2 biochemical enrichment. Transfected mimics of the MIR-28 siblings hsa-miR-28-5p (blue) and hsa-miR-708-5p (red) have similar read counts compared to other endogenous microRNAs (gray).

(**B**). Plot of genes with mRNA transcripts targeted by both hsa-miR-28-5p and hsa-miR-708-5p. The miR-eCLIP data were filtered for genes expressed in MCF10A cells, and RNAseq differential expression data following MIR-28 treatment is graphed for each gene on the x- and y-axes. Bolded points represent genes downregulated by both MIR-28 mimics by at least -0.5 $\log_2$ units, with a differential expression false discovery rate < 0.05 for both treatments. All genes are labeled with their HGNC symbol.

(**C**). Plot of genes with mRNA transcripts targeted exclusively (XOR) by either hsa-miR-28-5p or hsa-miR-708-5p. The miR-eCLIP data were filtered and graphed as above. The genes encoding mRNA transcripts were bound by hsa-miR-28-5p (blue) or bound by hsa-miR-708-5p (red), according to miR-eCLIP data. Select genes are labeled with HGNC symbol.

(**D-F**). Cumulative empirical distribution plots indicating RNAseq differential expression data for labeled groups of targets. Genes not targeted by MIR-28 siblings (black) are compared to genes targeted by hsa-miR-28-5p (**D**, blue line), by hsa-miR-708-5p (**E**, pink line), or by either MIR-28 sibling (**F**, maroon line) based on miR-eCLIP results. Data were analyzed by Kolmogorov-Smirnov testing in R.

Supplementary Figure 14

MYC CDS (NM_002467.6:397-490)

A hsa-miR-28-5p

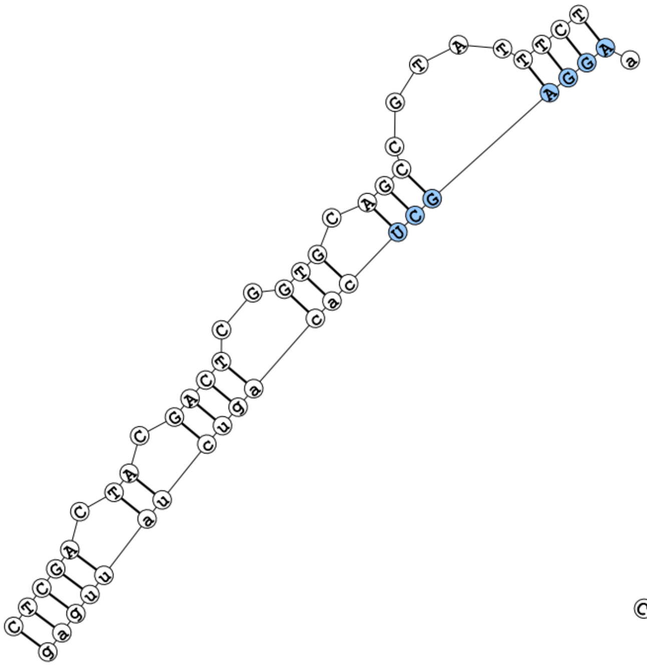

ENERGY = -13.0

B hsa-miR-708-5p

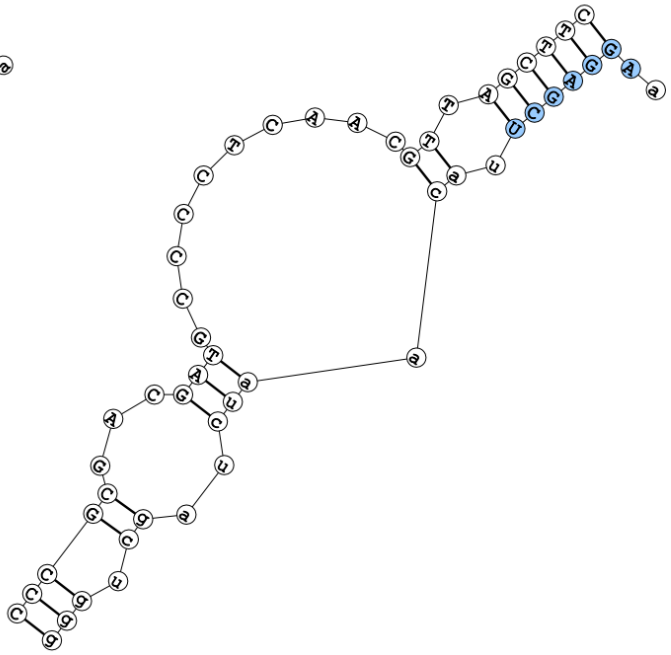

ENERGY = -12.7

CDKN1A 3’ UTR (NM_000389.5:1986-2103)

C hsa-miR-28-5p

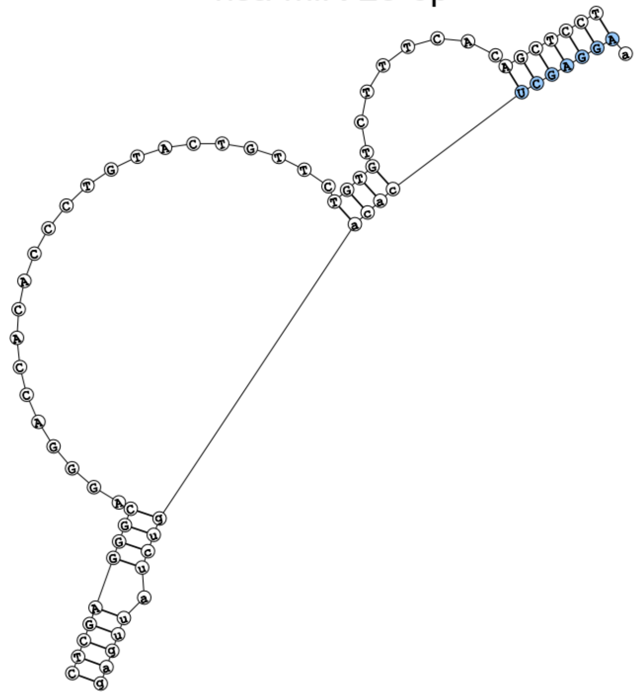

ENERGY = -16.6

D hsa-miR-708-5p

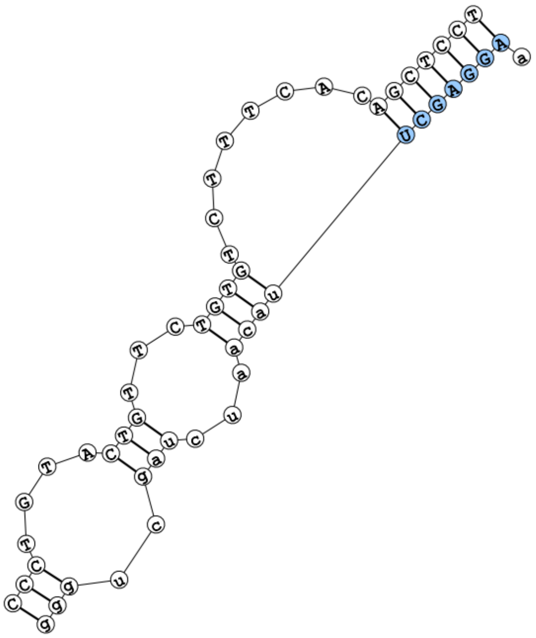

ENERGY = -15.3

Supplementary Figure 14. Predicted MIR-28 binding structures in the *MYC* CDS or *CDKN1A* (*p21*) 3’ UTR.

(**A-B**). DuplexFold predicted structures between hsa-miR-28-5p (**A**) or hsa-miR-708-5p (**B**) and wild-type (WT) putative MIR-28 sites in the *MYC* coding sequence (CDS). The seed sequence for each microRNA is shown in blue. The DuplexFold predicted binding energies are indicated for each structure. Transcriptomic coordinates for the binding region input are shown in the header. Extraneous target base pairs were removed for visualization.

(**C-D**). DuplexFold predicted structures between hsa-miR-28-5p (**C**) or hsa-miR-708-5p (**D**) and wild-type (WT) putative MIR-28 sites in *CDKN1A* (*p21*) 3’ UTR. Other features as in (**A-B**).
